# Causal relationship between gut microbiota and constipation: a bidirectional Mendelian randomization study

**DOI:** 10.3389/fmicb.2024.1438778

**Published:** 2024-07-17

**Authors:** Cuncheng Feng, Guanzhuang Gao, Kai Wu, Xiaoqi Weng

**Affiliations:** ^1^Department of Gastrointestinal Surgery, The Affiliated Changzhou No. 2 People's Hospital of Nanjing Medical University, Changzhou, China; ^2^Department of Gastrointestinal Surgery, Tongxiang First People's Hospital, Tongxiang, China

**Keywords:** constipation, gut microbiota, Mendelian randomization, GWAS, causal relationship

## Abstract

**Background:**

Constipation is a prevalent gastrointestinal disorder affecting approximately 15% of the global population, leading to significant healthcare burdens. Emerging evidence suggests that gut microbiota plays a pivotal role in the pathogenesis of constipation, although causality remains uncertain due to potential confounding factors in observational studies. This study aims to clarify the causal relationships between gut microbiota and constipation using a bidirectional Mendelian Randomization (MR) approach, which helps to overcome confounding issues and reverse causality.

**Methods:**

Utilizing data from genome-wide association studies (GWAS) from the MiBioGen consortium and other sources, we identified genetic variants as instrumental variables (IVs) for 196 bacterial traits and constipation. These IVs were rigorously selected based on their association with the traits and absence of linkage with confounding factors. We applied several MR methods, including Inverse Variance Weighted (IVW), MR Egger, and MR-PRESSO, to examine the causal effects in both directions.

**Results:**

Our analysis revealed a significant causal relationship where specific bacterial taxa such as *Coprococcus1* (OR = 0.798, 95%CI: 0.711–0.896, *p* < 0.001), *Coprococcus3* (OR = 0.851, 95%CI: 0.740–0.979, *p* = 0.024), *Desulfovibrio* (OR = 0.902, 95%CI: 0.817–0.996, *p* = 0.041), *Flavonifracto*r (OR = 0.823, 95%CI: 0.708–0.957, *p* < 0.001), and *Lachnospiraceae UCG004*, whereas others including *Ruminococcaceae UCG005* (OR = 1.127, 95%CI: 1.008–1.261, *p* = 0.036), *Eubacterium nodatum group* (OR = 1.080, 95%CI: 1.018–1.145, *p* = 0.025), *Butyricimonas* (OR = 1.118, 95%CI: 1.014–1.233, *p* = 0.002), and *Bacteroidetes* (OR = 1.274, 95%CI: 1.014–1.233, *p* < 0.001) increase constipation risk. In the reverse MR analysis, constipation was found to influence the abundance of certain taxa, including *Family XIII*, *Porphyromonadaceae*, *Proteobacteria*, *Lentisphaeria*, *Veillonellaceae*, *Victivallaceae*, *Catenibacterium*, *Sellimonas*, and *Victivallales*, indicating a bidirectional relationship. Sensitivity analyses confirmed the robustness of these findings, with no evidence of heterogeneity or horizontal pleiotropy.

**Conclusion:**

The relationship between our study gut microbiota and constipation interacts at the genetic level, which gut microbiota can influence the onset of constipation, and constipation can alter the gut microbiota. *Coprococcus1*, *Coprococcus3*, *Desulfovibrio*, *Flavonifractor* and *Lachnospiraceae UCG004* play a protective role against constipation, while *Ruminococcaceae UCG005*, *Eubacterium nodatum group*, *Butyricimonas*, and *Bacteroidetes* are associated with an increased risk. In addition, constipation correlates positively with the abundance of *Family XIII*, *Porphyromonadaceae* and *Proteobacteria*, while negatively with *Lentisphaeria*, *Veillonellaceae*, *Victivallaceae*, *Catenibacterium*, *Sellimonas*, and *Victivallales*.

## 1 Introduction

Constipation is an intestinal disorder based on symptoms such as difficult, infrequent or incomplete bowel movements, dry, hard stools, which affects around 15% of the global population (Mancabelli et al., 2017; Bharucha and Lacy, 2020; Hojo et al., 2023). In contemporary society, factors such as the accelerated pace of life, heightened societal pressures, and poor dietary choices have exacerbated the prevalence of this condition (Zhang X. et al., 2021; Hojo et al., 2023; Diaz et al., 2024). Long-term constipation not only reduces people’s quality of life, but also causes serious psychological damage (Wald et al., 2007; Yun et al., 2024). A study involving 12,352 participants in the United States revealed that individuals suffering from constipation were significantly more prone to major depression compared to those without constipation (Wang P. et al., 2023). The widespread occurrence of constipation, combined with its chronic nature and considerable effect on quality of life, leads to substantial utilization of healthcare resources (Rodriguez-Ramallo et al., 2021; Harris and Chang, 2022). The annual direct costs of managing constipation in the USA vary between $1,912 and $7,522 per patient (Nellesen et al., 2013).

In the United Kingdom, constipation is a significant health concern, resulting in more than one million consultations with general practitioners and approximately 69,000 hospitalizations annually (Shafe et al., 2011). Additionally, the National Health Service spent approximately £168 million on treating constipation during 2018–2019, which included expenses for prescribed laxatives and hospital admissions related to the condition. In Romania, the annual expenditure on laxatives, encompassing both prescribed and over-the-counter options, is estimated to be around 15 million euros (Albu et al., 2019). Current understanding indicates that constipation is caused by a range of contributing factors such as malfunctioning of the intestinal nervous system, visceral hypersensitivity, irregular distribution of interstitial cells of Cajal, and diminished gastrointestinal motility (Sikirov, 1989; Fu et al., 2021). Risk factors for developing constipation encompass older age, female gender, insufficient physical activity, low caloric intake, dietary habits, and alterations in intestinal flora (Mancabelli et al., 2017; Katsirma et al., 2021). Additionally, recent research indicates that an imbalance in gut microbiota, termed dysbiosis, is also a significant risk factor for constipation (Fu et al., 2021; Zhang S. et al., 2021).

The gastrointestinal tract hosts the gut microbiota, which comprises approximately 100 trillion bacteria spanning over 1,000 different species. This intestinal microecology plays a critical role in influencing the host’s metabolism, immune function, digestion, and overall development (Adak and Khan, 2019). Research has indicated variations in the composition of gut microbiota between individuals who are constipated and those who are not, indicating that the gut microbiota and its metabolites may be intricately linked with the development of constipation through complex interactions (Yang et al., 2022). However, significant differences exist in the results of many studies. For instance, Kim et al. (2015) studied the fecal flora of patients with functional constipation using a real-time quantitative polymerase chain reaction method and reported a decreased concentration of *bifidobacteria* and *lactobacilli* in people with constipation. Zhu et al. (2014) and Yarullina et al. (2020) conducted a preliminary cross-sectional study employing 16S rRNA sequencing and discovered that the levels of *Bifidobacterium* spp. and *Lactobacillus* spp. remained unchanged in adolescents with obesity-related constipation as well as in patients with severe constipation. Additionally, in elderly patients suffering from chronic constipation (CC), the abundance of *Bacteroides* was found to be significantly higher compared to healthy controls (Guo et al., 2020).

These studies are controversial, likely due to the following reasons: firstly, the different resolution capabilities of culture-based versus molecular methods in analyzing the gut microbiota; secondly, some studies indirectly assess differences between gut flora by detecting the flora in feces; thirdly, variations in the sources of samples for clinical studies and confounding biases in the studies themselves; and finally, there is a notable lack of an ideal animal model that accurately reflects the anatomical and functional abnormalities associated with constipation. Moreover, it remains unclear whether changes in the gut flora cause constipation or if constipation itself induces changes in the gut flora. Therefore, developing a new methodology to address these shortcomings is urgent.

Mendelian randomization (MR) analysis employs genetic variants, including Single Nucleotide Polymorphisms (SNPs), as instrumental variables, adhering to Mendel’s law of independent assortment (Sekula et al., 2016). This method treats genetic allocation at conception as similar to random assignment in controlled trials, helping observational studies overcome issues like residual confounding and reverse causality, thereby boosting their credibility (Birney, 2022). Our study employs MR analysis to explore the causal relationship between gut microbiota and constipation, aiming to clarify the pathogenesis and improve treatment approaches. This research seeks to deepen the understanding of how gut microbiota influences constipation.

## 2 Methods

### 2.1 Study design

**Figure 1:**
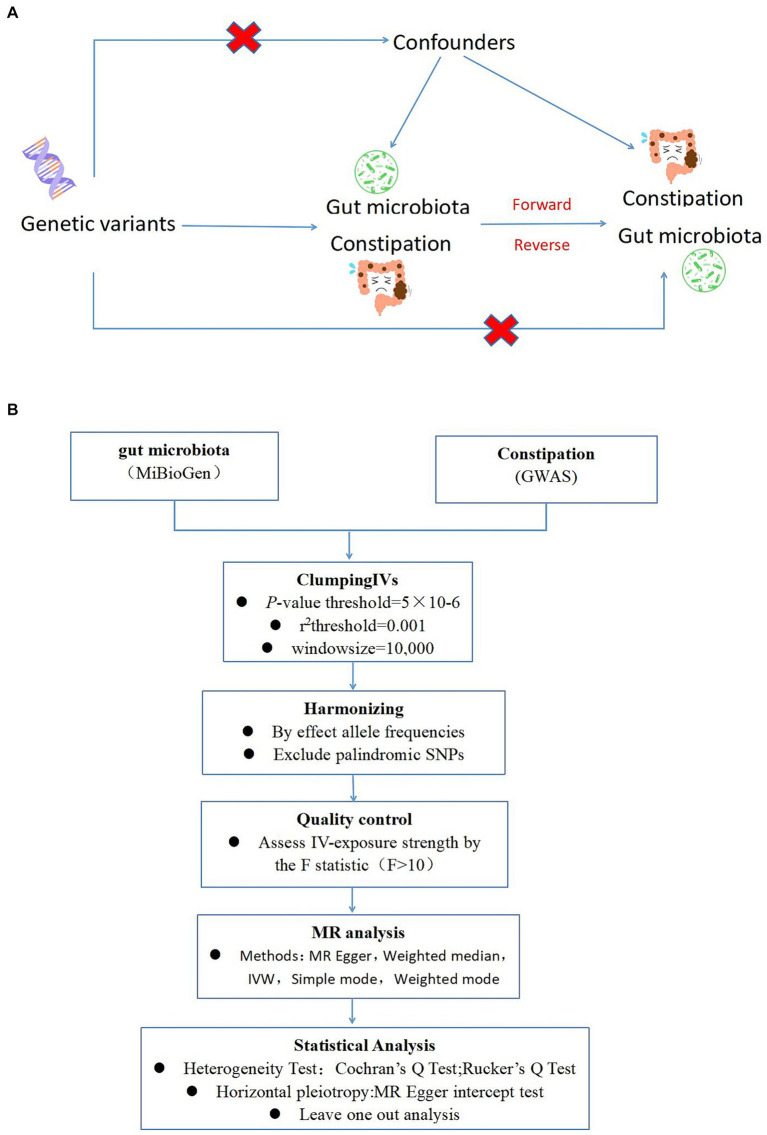
**(A)** A schematic diagram illustrates the MR causality study design, elucidating the fundamental principles of MR study and the hypothetical relationship between genetic variant, exposure, and outcome. **(B)** A Schematic model of the Mendelian randomization (MR) study. GWAS, Genome Wide Association Studies; IV, Instrumental variable; SNP, single nucleotide polymorphism; MR, Mendelian randomization; IVW, Inverse-variance weighted.

Drawing on the genome-wide association study (GWAS) summary data for gut microbiota and constipation, this investigation carefully selected eligible instrumental variables (IVs) for Mendelian Randomization (MR) analysis to delineate the causal dynamics between gut microbiota and constipation (Burgess et al., 2017). The methodology rigorously adhered to the tripartite foundational assumptions of MR analysis: (1) The IVs identified had a direct association with the exposure variable; (2) The IVs were not associated with any confounding factors, ensuring their independence; (3) The IVs exerted influence on the outcome solely through their interaction with the exposure variable (Figure 1A). The datasets deployed in this research are publicly accessible, with ethical approval and written informed consent having been secured during the preliminary investigations. Thus, additional ethical approvals are no longer required.

### 2.2 Data source

GWAS summary data for gut microbiota were sourced from the MiBioGen consortium website,^1^ encompassing 18,340 samples of European descent (Birney, 2022). A total of 196 bacterial traits (classified into specific phylum, class, order, family, and genus) were obtained, with the sample size reaching 14,306. Since 15 bacterial traits lacked specific species names, we excluded them and selected 196 bacterial traits for analysis. The constipation data was derived from the GWAS database. We analyzed summary-level data from 411,623 European individuals, which included 24,176,599 SNPs.

### 2.3 IV selection

To ensure the robustness and reliability of our Mendelian Randomization (MR) analysis, we implemented stringent quality controls for instrumental variable (IV) selection, adhering to the three foundational assumptions of MR analysis (Figure 1). Initially, we identified SNPs associated with 212 gut microbiota entities with a significance threshold of *p* < 1E-5. To mitigate the influence of linkage disequilibrium (LD), SNPs within strong LD were excluded (*r*^2^ < 0.001, clumping distance = 10,000 kb). Furthermore, only SNPs with an F-statistic >10 were selected to satisfy the criterion for a strong association with the exposure. The F-statistic was calculated using the formula: F = β^2^exposure/SE^2^exposure (Kurilshikov et al., 2021), to assess the robustness of the instrumental SNPs, considering an F-statistic >10 indicative of minimal weak instrument bias. Additionally, palindromic SNPs with intermediate allele frequencies were removed to enhance the accuracy of the results (Figure 1B).

### 2.4 Statistical analysis

Our MR analysis was conducted using five distinct approaches: the random-effects inverse variance weighted (IVW) method as the primary analysis, complemented by MR Egger, weighted median, simple mode, and weighted mode analyses. The random-effects IVW results served as the cornerstone of our study. To evaluate heterogeneity, we utilized Cochran’s Q statistic for MR-IVW and Rucker’s Q statistic for MR Egger, with *p*-values >0.05 indicating no significant heterogeneity (Guo et al., 2020). The MR Egger intercept test was employed to assess horizontal pleiotropy, with *p*-values >0.05 suggesting an absence of horizontal pleiotropy. Moreover, the MR-PRESSO test not only identified horizontal pleiotropy but also detected outliers. The “Leave one out” analysis was instrumental in determining if a single SNP disproportionately influenced the causal relationship between gut microbiota and constipation. The global test in MR-PRESSO analysis was applied for horizontal pleiotropy assessment, and the distortion test within the same framework was utilized to ascertain the presence of outliers in our MR analysis. In addition, we performed reverse Mendelian analyses on several statistically significant positive intestinal flora screened to rule out reverse causality. All Mendelian Randomization analyses were performed utilizing the “Two Sample MR” (version 0.5.7) and “MR-PRESSO” (version 1.0) packages in R version 4.3.3, setting statistical significance at *p* < 0.05.

## 3 Results

### 3.1 MR analysis

**Figure 2:**
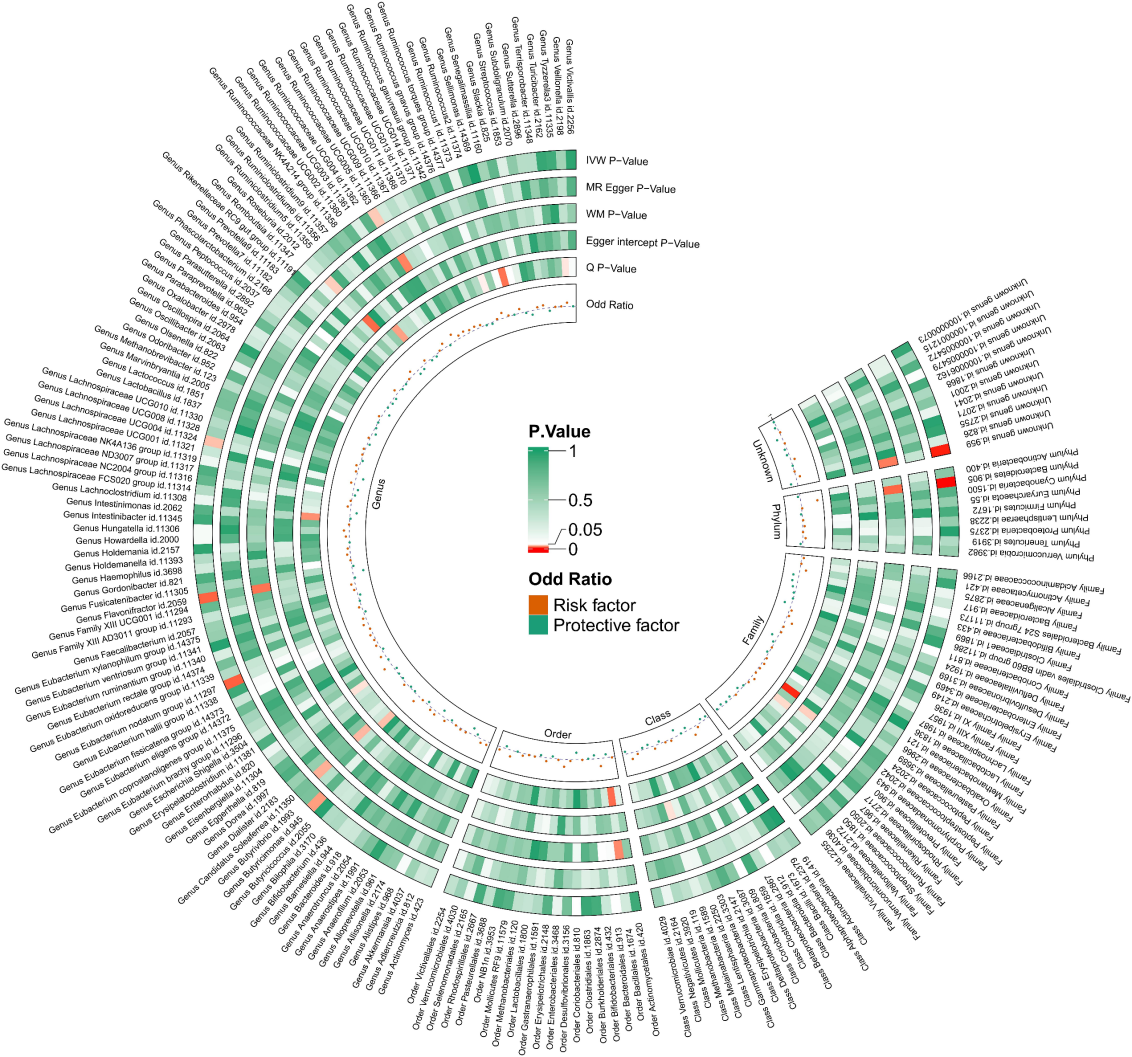
212 species of gut microbiota for their causal association with constipation.

**Figure 3:**
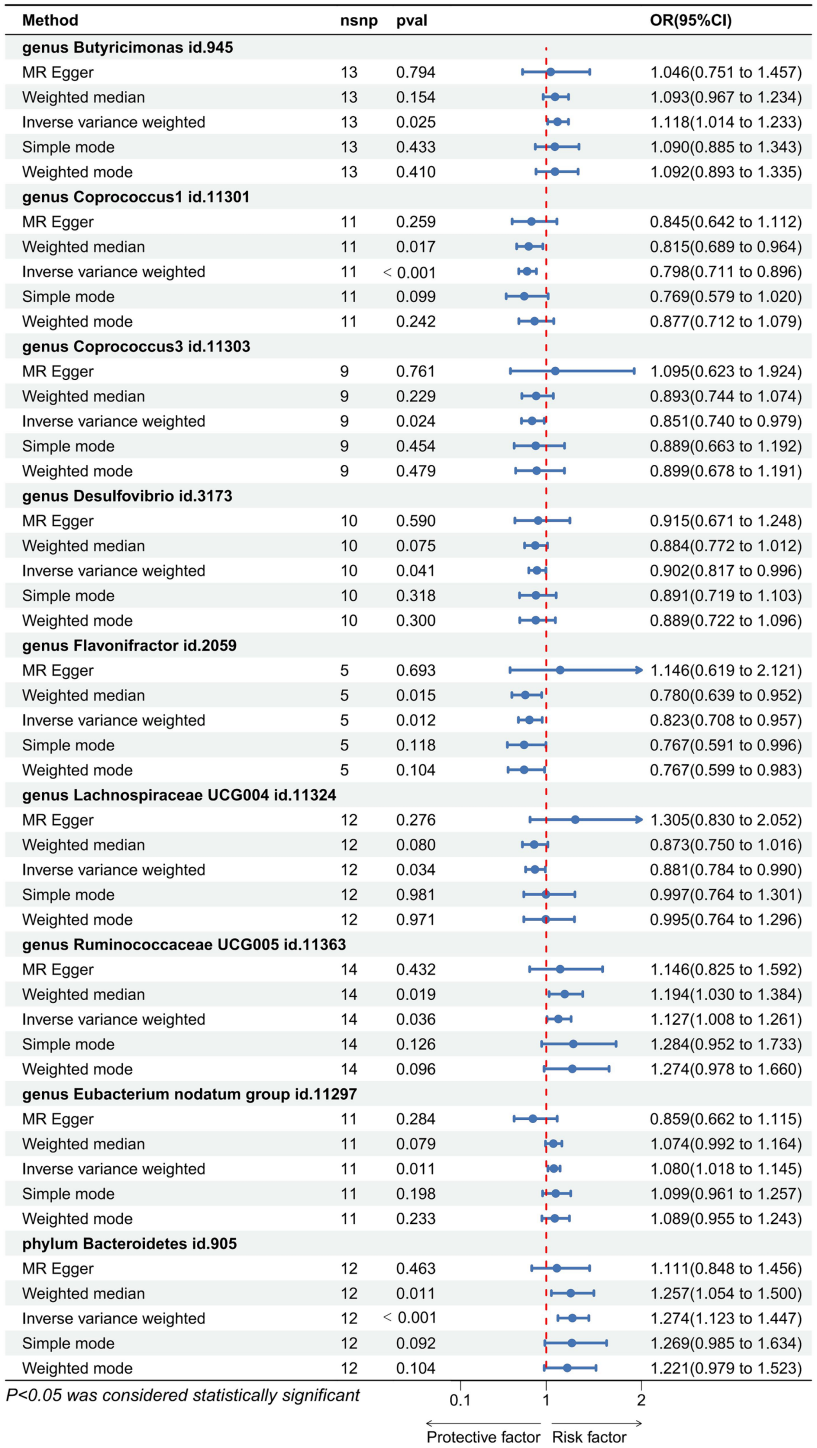
Forest plot showing the causal relationship between the genetically identified 9 microbial taxa and constipation using the MR analysis. The blue line segments and blue dots indicate the 95% CIs and OR-value for the different gut microbiota for the 5 methods (IVW, MR Egger, Weighted median, Simple mode, and Weighted mode).

Utilizing the IVW method as our primary analytical approach, 196 intestinal microbiota have been used as exposure and constipation as an outcome. We analyzed the causal relationship between 196 gut microbiota and constipation (Figure 2), and established a causal relationship between the genetically predicted relative abundance of nine bacterial taxa and constipation, as detailed in Figure 3. Our IVW analysis revealed that certain bacteria displayed a protective effect against constipation. These include *Coprococcus1*, with an odds ratio (OR) of 0.798 and a 95% confidence interval (CI) of 0.711–0.896 (*p* < 0.001), *Coprococcus3* (OR = 0.851, 95%CI: 0.740–0.979, *p* = 0.024), *Desulfovibrio* (OR = 0.902, 95%CI: 0.817–0.996, *p* = 0.041), *Flavonifractor* (OR = 0.823, 95%CI: 0.708–0.957, *p* < 0.001), and *Lachnospiraceae UCG004* (OR = 0.881, 95%CI: 0.784–0.990, *p* = 0.034). Conversely, other taxa were associated with an increased risk of constipation. These include *Ruminococcaceae UCG005* (OR = 1.127, 95%CI: 1.008–1.261, *p* = 0.036), *Eubacterium nodatum group* (OR = 1.080, 95%CI: 1.018–1.145, *p* = 0.025), *Butyricimonas* (OR = 1.118, 95%CI: 1.014–1.233, *p* = 0.002), and *Bacteroidetes* (OR = 1.274, 95%CI: 1.014–1.233, *p* < 0.001), indicating a contributory role in constipation (Figure 3).

### 3.2 Reverse-direction Mendelian randomization analysis

**Figure 4:**
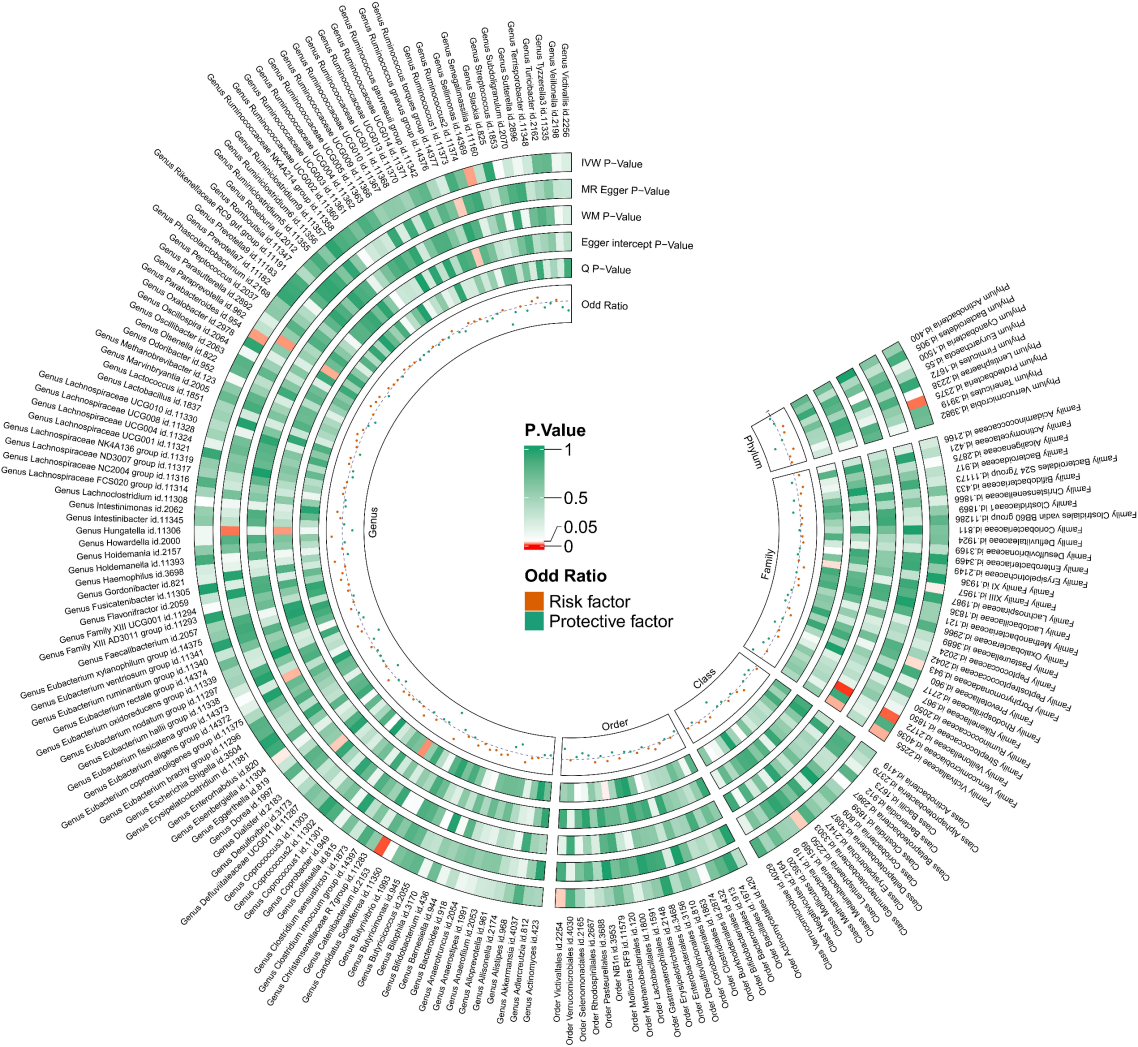
Causal relationship between constipation and 212 gut microorganisms.

**Figure 5:**
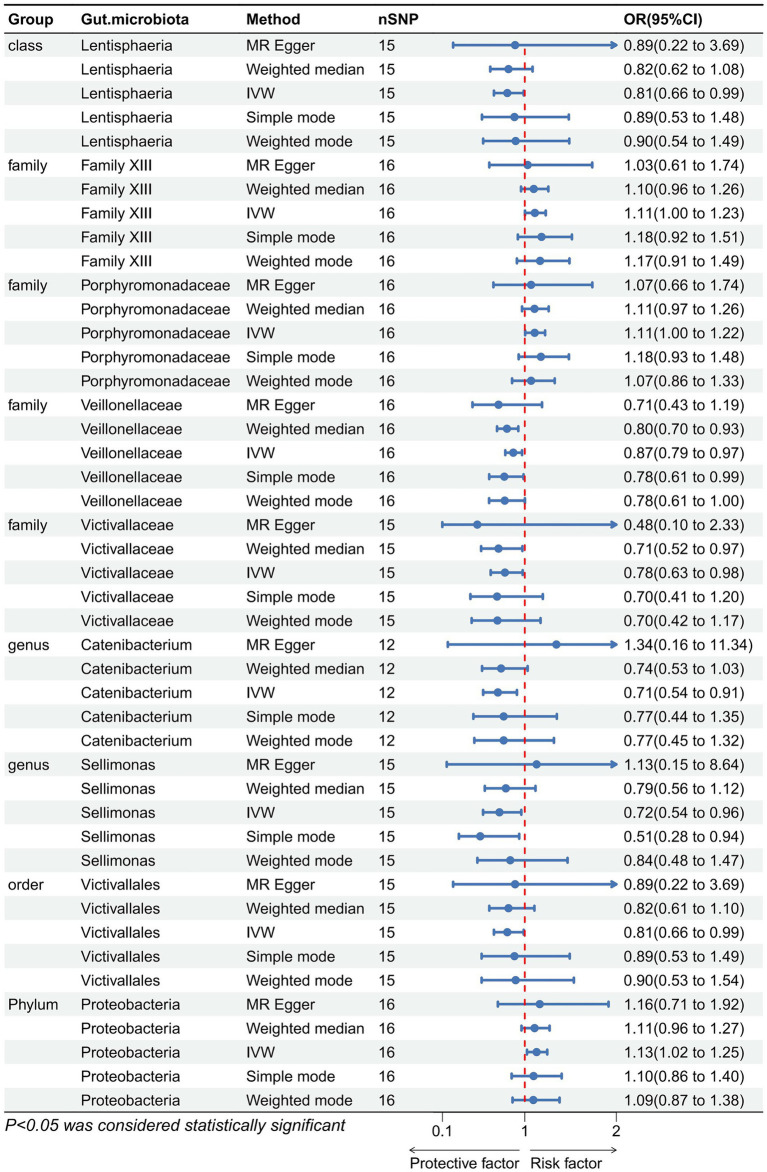
Forest plot showing reverse Mendelian randomization to study the existence of a causal relationship between constipation and nine gut microbiota. The blue line segments and blue dots indicate the 95% CIs and OR-value for the different gut microbiota for the 5 methods (IVW, MR Egger, Weighted median, Simple mode, and Weighted mode).

We performed an inverse Mendelian randomization analysis using the same methodology with constipation as the exposure and gut microbiota in 196 as the outcome (Figure 4). The analysis flow is shown in Figure 1B. The IVW analysis showed a positive correlation between constipation and three gut microbiota, while a negative correlation with six gut microbiota. Specifically, IVW results showed that constipation leads to increased abundance of *Family XIII* (OR = 1.110, 95%CI: 1.002–1.229, *p* = 0.046), *Porphyromonadaceae* (OR = 1.108, 95%CI: 1.004–1.222, *p* = 0.041), *Proteobacteria* (OR = 1.129, 95%CI: 1.024–1.245, *p* = 0.015). Conversely, Constipation causes a decrease in the abundance of *Lentisphaeria* (OR = 0.809, 95%CI: 0.663–0.988, *p* = 0.037), *Veillonellaceae* (OR = 0.874, 95%CI: 0.787–0.970, *p* = 0.011), *Victivallaceae* (OR = 0.783, 95%CI: 0.628–0.977, *p* = 0.030), *Catenibacterium* (OR = 0.706, 95%CI: 0.545–0.914, *p* = 0.008), *Sellimonas* (OR = 0.723, 95%CI: 0.544–0.962, *p* = 0.026), and *Victivallales* (OR = 0.809, 95%CI: 0.663–0.988, *p* = 0.037) (Figure 5).

### 3.3 Sensitivity analyses

Cochran’s Q statistic and Rucker’s Q statistic analyses in our Mendelian Randomization (MR) study indicated no significant heterogeneity across the MR analyses of *Coprococcus1*, *Coprococcus3*, *Desulfovibrio*, *Flavonifractor*, *Lachnospiraceae UCG004*, *Ruminococcaceae UCG005*, *Eubacterium nodatum group*, *Butyricimonas*, and *Bacteroidetes* in relation to constipation, with all *p*-values exceeding 0.05. Furthermore, the MR Egger intercept test revealed no evidence of horizontal pleiotropy for all examined taxa (*p* > 0.05), as detailed in Table 1. The robustness of our findings was further validated by the “Leave one out” analysis, which demonstrated that no single SNP disproportionately affected the causal inference.

**Table 1:** Sensitivity analyses were performed on the results of the forward MR analyses of exposure and outcome.

Exposure	Outcome	Heterogeneity test		Pleiotropy test	MR-PRESSO	
		Cochran’s Q test (*p* value)	Rucker’s Q test (*p* value)	Egger intercept (*p* value)	Distortion test	Global test
		IVW	MR-Egger	MR-Egger	Outliers	*p* Value
*Coprococcus1*	Constipation	0.635	0.561	0.669	NA	0.690
*Coprococcus3*	Constipation	0.736	0.735	0.359	NA	0.810
*Desulfovibrio*	Constipation	0.669	0.570	0.927	NA	0.700
*Flavonifractor*	Constipation	0.838	0.969	0.356	NA	0.790
*Lachnospiraceae UCG004*	Constipation	0.899	0.990	0.109	NA	0.890
*Ruminococcaceae UCG005*	Constipation	0.290	0.227	0.917	NA	0.300
*Eubacterium nodatum group*	Constipation	0.652	0.863	0.112	NA	0.700
*Butyricimonas*	Constipation	0.975	0.962	0.689	NA	0.970
*Bacteroidetes*	Constipation	0.928	0.956	0.286	NA	0.960

Additionally, the MR-PRESSO global test confirmed the absence of horizontal pleiotropy across all examined taxa (*p* > 0.05), and the distortion test verified that there were no outliers influencing the results of our MR analyses (Table 2). In the inverse Mendelian Randomization analysis focusing on the relationship of constipation with gut microbiota, similar results were observed, showing no significant heterogeneity or horizontal pleiotropy, thus supporting the consistency and reliability of our causal interpretations (Table 2).

**Table 2:** Sensitivity analyses were performed on the results of the reverse MR analyses of exposure and outcome.

Exposure	Outcome	Heterogeneity test		Pleiotropy test	MR-PRESSO	
		Cochran’s Q test (*p* Value)	Rucker’s Q test (*p* Value)	Egger intercept (*p* Value)	Distortion test	Global test
		IVW	MR-Egger	MR-Egger	Outliers	*p* Value
Constipation	*Lentisphaeria*	0.493	0.417	0.891	NA	0.450
Constipation	*Family XIII*	0.883	0.844	0.781	NA	0.870
Constipation	*Porphyromonadaceae*	0.922	0.888	0.884	NA	0.900
Constipation	*Veillonellaceae*	0.535	0.508	0.439	NA	0.540
Constipation	*Victivallaceae*	0.432	0.385	0.552	NA	0.420
Constipation	*Catenibacterium*	0.897	0.873	0.564	NA	0.920
Constipation	*Sellimonas*	0.134	0.106	0.672	NA	0.160
Constipation	*Victivallales*	0.493	0.417	0.891	NA	0.510
Constipation	*Proteobacteria*	0.452	0.380	0.908	NA	0.500

## 4 Discussion

In our study, we employed MR methodology to investigate the genetic causal relationships between nine specific gut microbiota taxa and constipation. This genetic-based causal analysis helps us bypass common constraints found in traditional observational studies. We discovered that *Coprococcus1*, *Coprococcus3*, *Desulfovibrio*, *Flavonifractor*, and *Lachnospiraceae UCG004* appear to play a protective role against constipation, while *Ruminococcaceae UCG005*, *Eubacterium nodatum group*, *Butyricimonas*, and *Bacteroidetes* are associated with an increased risk. Furthermore, our findings indicate that constipation correlates positively with the abundance of *Family XIII*, *Porphyromonadaceae*, and *Proteobacteria*, and negatively with *Lentisphaeria*, *Veillonellaceae*, *Victivallaceae*, *Catenibacterium*, *Sellimonas*, and *Victivallales*.

*Coprococcus1* and *Coprococcus3*, belonging to the phylum *Clostridium*, produce butyrate, a short-chain fatty acid (SCFA). Research has shown that SCFAs, like butyrate, are pivotal in modulating intestinal peristalsis and enhancing gut barrier integrity through the upregulation of tight junction proteins or mucins. This elucidates the crucial role that microbial metabolites play in intestinal health and suggests that boosting the production of beneficial SCFAs may offer a therapeutic strategy for managing constipation. This in-depth analysis enhances our comprehension of how genetic predispositions and gut microbiota configurations impact constipation, significantly broadening our understanding of the microbiome’s role in gastrointestinal health (Wang L. et al., 2023). In addition, previous research has highlighted that butyrate-producing flora can enhance colonic motility and alleviate constipation by inducing the release of serotonin or by stimulating cholinergic pathways through butyrate production. This mechanism underscores the critical role of butyrate as a key biochemical mediator in gastrointestinal health (Fukui et al., 2018). In a clinical cohort study involving 68 functionally constipated individuals and 79 healthy controls, Mancabelli et al. (2017) observed that the constipated group exhibited a significantly lower abundance of *Coprococcus3* bacteria compared to the control group. This aligns with our findings. Intriguingly, however, *Butyricimonas*, another butyric acid-producing bacteria, presented the opposite effect in our study, emerging as a risk factor for constipation. Our analysis suggests that these conflicting outcomes may be attributed to varying concentrations of butyrate produced by different gut flora. It has been documented that while high concentrations of butyrate may inhibit intestinal peristalsis, lower concentrations tend to enhance it. This differential impact highlights the complex role that butyrate concentrations play in regulating gut motility (Neunlist et al., 1999; Yarullina et al., 2020). Guo et al. (2020) analyzed fecal samples from 61 constipated patients and 48 age-matched healthy volunteers using 16S rRNA gene sequencing, discovering that the abundance of *Butyricimonas* in the intestinal flora of constipated patients was significantly higher compared to that of the healthy controls. Similarly, Mancabelli et al. (2017) observed a significantly higher abundance of *Butyricimonas* in a cohort of 25 patients with chronic constipation compared to 25 controls. These findings lend support to our study, reinforcing the complex relationship between *Butyricimonas* levels and constipation.

*Desulfovibrio* and *Flavonifractor* both produce hydrogen sulfide (H_2_S), a common metabolite crucial in the pathogenesis of constipation. The balance of hydrogen (H_2_), methane (CH_4_), and hydrogen sulfide (H_2_S)—byproducts of microbiota fermentation—plays a significant role in this condition. Methane, generated by gut microbiota, functions as a neuromuscular transmitter that affects gut motility. An *ex vivo* study demonstrated that methane exposure reduced contractility in ileal muscles. Conversely, hydrogen infusion not only increased ileal muscle contractility but also led to a reduction in colonic transit time. In the gastrointestinal tract, H_2_S-producing bacteria compete with methanogens for hydrogen (Smith et al., 2019; Villanueva-Millan et al., 2022). Our findings suggest that *Desulfovibrio* and *Flavonifractor* act as protective factors against constipation, potentially due to their competition for hydrogen with methanogenic bacteria. This hypothesis requires further experimental verification. A randomized controlled trial (RCT) demonstrated that targeting methanogenic bacteria in the gut with antibiotic treatment to reduce methane production could accelerate colonic transit and consequently improve symptoms of constipation (Ghoshal et al., 2018). Similarly, the effect of *Ruminococcaceae UCG005* on promoting constipation may also be related to methane production. Although there are no direct studies indicating that *Ruminococcaceae UCG005* produces methane, it is known to be in a symbiotic relationship with methanogenic bacteria (Djemai et al., 2022; Villanueva-Millan et al., 2022). Therefore, *Ruminococcaceae UCG005* may influence constipation by enhancing methane production through its symbiotic methanogenic bacteria.

*Bacteroidetes*, constituting approximately 25% of the total intestinal bacteria in adults, play a crucial role in the development and maintenance of sensory and motor functions in the intestine (Hooper et al., 2001; Barbara et al., 2005). Specifically, the genus *Bacteroides* is recognized for its role in influencing intestinal motility through the enhancement of the expression of γ-aminobutyric acid (GABA), vesicle-associated protein-33 (VAP-33), and enteric γ-actin (Bhattarai et al., 2018; Costa et al., 2022). Additionally, the metabolism of bile acids (BAs) is essential for managing constipation-related symptoms. *Bacteroidetes* influence this process by stimulating colonic motility through alterations in the bile acid pool composition (Duan et al., 2023). Our study indicates that a high abundance of *Bacteroidetes* is a risk factor for constipation. This observation is supported by previous research. For instance, Fan et al. (2022) noted a significant increase in *Bacteroidetes* abundance in constipated patients through 16S rRNA metagenomic profiling of intestinal flora from 30 individuals. Similarly, Wang J. et al. (2023) reported in a clinical study that patients with functional constipation exhibited significantly higher levels of *Bacteroidetes* compared to a healthy cohort. These findings highlight the complex role of *Bacteroidetes* in gastrointestinal health and their potential impact on constipation. In addition, the intestinal microbiota and its metabolites play a crucial role in inflammation and the immune regulatory system, significantly affecting intestinal motility and the development of constipation. Increasing evidence indicates that disturbances in the gut microbial community can lead to the proliferation of otherwise low-abundance, harmful bacteria, which in turn exacerbate intestinal inflammation. This pro-inflammatory state often results in weakened intestinal peristalsis, thereby contributing to the onset of constipation. For instance, an expansion of *Enterobacteriaceae* is frequently observed in cases of gut dysbiosis associated with various forms of intestinal inflammation. Moreover, studies have demonstrated that patients with constipation exhibit increased numbers of CD8+, CD4+, CD3+, and CD25+ T cells, as well as enhanced lymphocyte proliferation, indicating the activation of T cell-mediated immunity.

Our study uniquely discovered at the genetic level that *Lachnospiraceae UCG004* acts as a protective factor against constipation, while the *Eubacterium nodatum group* is a risk factor, findings that have seldom been reported in previous research. Further investigation is required to confirm these results. Interestingly, numerous clinical studies have demonstrated that *Lactobacillus* and *Bifidobacterium*, well-known probiotics, help alleviate bowel difficulties in patients with constipation and act as protective factors (Dimidi et al., 2017; Fuyuki et al., 2021; Wang et al., 2022; Lai et al., 2023). However, our research did not establish a direct genetic causal link between these probiotics and constipation (Figure 4).

In fact, this controversy has persisted in recent years. Chassard et al. (2012) found significantly lower abundance of *Lactobacillus* and *Bifidobacterium* in the intestinal flora of constipated patients by bacterial culture in a comparison of 14 female constipated patients and a healthy control population. Yarullina et al. (2020) studied the gut microbiota in colon tissue samples from 20 patients with refractory chronic constipation using culture and 16S rRNA macrogenomic analyses and found no significant changes in the abundance of *Bifidobacteria* and *Lactobacilli* in the patients with constipation as compared to controls. These differences in results may be due to differences in sample sources and research methods. We support the role of *Bifidobacterium* and *Lactobacillus* in improving constipation symptoms, but are cautious about the idea that *Bifidobacterium* and *Lactobacillus* have a direct causal relationship with constipation.

Our reverse Mendelian randomization analysis indicates that constipation can lead to changes in the abundance of certain intestinal flora. Mancabelli et al. (2017) conducted a 16S rRNA-based microbial profiling analysis on 147 stool samples from 68 individuals with functional constipation and compared their microbial profiles to those of 79 healthy subjects. The results indicated a significantly higher abundance of *Porphyromonadaceae* in the intestinal flora of patients with functional constipation. This is consistent with our findings. However, Wang J. et al. (2023) utilized the 16S rRNA method to analyze the gut microbiota of 28 patients with functional constipation and 30 healthy individuals, and discovered that constipated patients exhibited a lower abundance of *Proteobacteria*. This result contradicts our findings. The discrepancies between these studies could be attributed to differences in research methods, sample sources, and dietary habits.

For the first time, we employed bidirectional Mendelian randomization to study the causal relationship between gut flora and constipation. Our findings indicate that the interaction between gut flora and constipation is reciprocal: gut flora can both promote or ameliorate constipation, and conversely, constipation can also lead to specific changes in the gut microbiota at the genetic level. These insights open up new avenues for the development of innovative diagnostic tools and potential treatments. To further understand the role of altered microbiota in the pathogenesis of changes in colonic motility, additional experimental or preclinical studies are necessary (Tian et al., 2021). These findings highlight the dynamic interplay between our genetic makeup and gut microbiota, underscoring the complexity of gastrointestinal health.

Definitely, this study has its limitations. It primarily focuses on a European population, and thus, caution is warranted when extending these findings to other ethnic groups. Additionally, since constipation is influenced by multiple factors, genetic contributions represent only a portion of its overall pathogenesis. The impacts of dietary habits and living conditions on the causal relationship between constipation and gut microbiota also require further exploration. Moreover, the study relies on publicly available summary statistics from GWAS for examining outcomes and exposure characteristics. This reliance on summary statistics rather than individual-level data restricts further subgroup analyses and limits the generalizability of the findings across different populations and taxonomic levels. Future research should delve into the mechanisms through which intestinal flora and its metabolites contribute to constipation. Nonetheless, this study offers novel genetic perspectives on the relationship between gut microbiota and constipation, setting the stage for additional investigations in this area. Meanwhile, our findings have significant guidance for the clinical management of constipation. Based on our research, new probiotic agents as well as antibiotics targeting harmful bacteria may be developed, thus reducing the burden of patients suffering from constipation all over the world.

## 5 Conclusion

The relationship between our study gut microbiota and constipation interacts at the genetic level, which gut microbiota can influence the onset of constipation, and constipation can alter the gut microbiota. *Coprococcus1*, *Coprococcus3*, *Desulfovibrio*, *Flavonifractor*, and *Lachnospiraceae UCG004* play a protective role against constipation, while *Ruminococcaceae UCG005*, *Eubacterium nodatum group*, *Butyricimonas*, and *Bacteroidetes* are associated with an increased risk. In addition, constipation correlates positively with the abundance of *Family XIII*, *Porphyromonadaceae*, and *Proteobacteria*, and negatively with *Lentisphaeria*, *Veillonellaceae*, *Victivallaceae*, *Catenibacterium*, *Sellimonas*, and *Victivallales*.

## Data availability statement

The datasets presented in this study can be found in online repositories. The names of the repository/repositories and accession number(s) can be found in the article/supplementary material.

## Author contributions

CF: Conceptualization, Data curation, Methodology, Writing – original draft. GG: Methodology, Software, Supervision, Writing – review & editing. KW: Data curation, Formal analysis, Methodology, Software, Writing – review & editing. XW: Data curation, Formal analysis, Funding acquisition, Methodology, Project administration, Supervision, Validation, Writing – review & editing.

## Footnotes

^1^ www.mibiogen.org
